# Evidence-based investment selection: Prioritizing agricultural development investments under climatic and socio-political risk using Bayesian networks

**DOI:** 10.1371/journal.pone.0234213

**Published:** 2020-06-05

**Authors:** Barbaros Yet, Christine Lamanna, Keith D. Shepherd, Todd S. Rosenstock

**Affiliations:** 1 Hacettepe University, Ankara, Turkey; 2 World Agroforestry (ICRAF), Nairobi, Kenya; 3 CGIAR Research Program on Water, Land and Ecosystems, Nairobi, Kenya; 4 World Agroforestry (ICRAF), Kinshasa, Democratic Republic of Congo; 5 CGIAR Research Program on Climate Change, Agriculture and Food Security, Kinshasa, Democratic Republic of Congo; Queen Mary University of London, UNITED KINGDOM

## Abstract

Agricultural development projects have a poor track record of success mainly due to risks and uncertainty involved in implementation. Cost-benefit analysis can help allocate resources more effectively, but scarcity of data and high uncertainty makes it difficult to use standard approaches. Bayesian Networks (BN) offer a suitable modelling technology for this domain as they can combine expert knowledge and data. This paper proposes a systematic methodology for creating a general BN model for evaluating agricultural development projects. Our approach adapts the BN model to specific projects by using systematic review of published evidence and relevant data repositories under the guidance of domain experts. We evaluate a large-scale agricultural investment in Africa to provide a proof of concept for this approach. The BN model provides decision support for project evaluation by predicting the value—measured as net present value and return on investment—of the project under different risk scenarios.

## 1 Introduction

Billions of US dollars are being invested to address climate change by international finance institutions, national governments, and the private sector. At the United Nations Framework Convention on Climate Change Conference of Parties in Poland (COP24), the World Bank pledged 200 billion USD to fund climate resilience over the next five years [1]. The International Fund for Agricultural Development (IFAD) leads one of the largest adaptation-focused funds of nearly 400 million USD [2]. This, however, is only a small fraction of the finance required. Nearly 90 trillion US dollars will be needed to meet the 2 degrees Celsius target that would reduce the risk of catastrophic impacts of climate change [3].

Much of the investment in developing countries, especially of Africa, is directed toward agriculture and land use. This is because agriculture represents a significant fraction of gross domestic product and employment of developing countries [4]. Also, the context of agricultural production where farmers have little access to inputs or assets to adapt to hazards makes this group among the most vulnerable to future climate change and variability [5]. African governments have also made commitments to support agricultural development. Forty-seven countries have signed the Comprehensive African Agriculture Development Program (CAADP) which sets the targets of 6% annual growth in agricultural GDP and at least 10% of public expenditures to the agricultural sector.

However, agricultural development investments have a poor track record of success. An evaluation of 86 agricultural project evaluations by the World Bank found that as many as 41% had non-positive outcomes [6]. This has significant implications: when benefits fall short of costs on projects financed on borrowed finance, the standard of living in the country declines. A variety of political, environmental and financial factors can affect the investment outcome, and these factors are often highly uncertain and interrelated. Data needed to predict outcome *ex-ante* are often scarce. When data are available, they are often relevant to only one of many factors that affect project outcomes, and this limits the use of purely data driven learning and evaluation approaches in this domain [7]. The complexity of the agricultural development environment, coupled with data scarcity, results in significant uncertainties for prioritization of investments and consequently reduced interest by financiers in agricultural investment.

Ex-ante predictions are typically based on cost-benefits analyses. However, the World Bank has expressed alarm over the fact that the percentage of their projects that are justified by cost-benefit analysis has been declining for several decades. This is attributed to a decline in adherence to standards and to difficulty in applying cost-benefit analysis [8]. Furthermore, standard approaches often assume that everything will go as planned. When integrated in the models, uncertainty is typically handled with partial or simulation-based sensitivity analyses that change the value of individual parameters, or uses Monte Carlo (MC) simulation [9]. However, partial sensitivity analyses disregard the joint effect of changing multiple parameters, and simulation-based sensitivity analyses have limited inference capabilities and interpretability of modelling assumptions [10,11]. Therefore, there is a need to develop new approaches that can handle both the multitude of driving forces, uncertainty and lack of data.

Bayesian Networks (BNs) are powerful modelling tools for dealing with such uncertainty when data are scarce. A BN is composed of a graphical structure that encodes the causal and associational relations associated with the problem. This enables experts to model domain knowledge about the causal relations in the BN. Parameters representing the strength of these relations can be defined based on data, published statistics, expert knowledge or in combination of these resources [12–14]. Once the BN is constructed for a problem, inference algorithms are available to compute probabilistic inference based on this structure and parameters. Despite these advantages, use of BN in investment decision making for agricultural development is limited. A major barrier for wider use of BNs in data-scarce domains is the difficulty of defining an appropriate BN structure and a probability distribution for each variable in the structure. Publications with realistic BN models often given a limited description of how they have built the model. Methods to assist BN construction with expert knowledge are either domain-specific [15,16] or require considerable modelling expertise to use in a specific domain [17,18]. Moreover, these methods are often focused on development of BN structure and assume sufficient data are available to learn the probability distributions, which is often not the case for agricultural development projects.

This paper presents a methodology for creating a general model for evaluating agricultural development projects under climatic and socio-political risks. Our method systematically guides the analyst in defining the impact, cost, and risk factor parameters regarding the project. These estimates are then used to define the probability distributions of the underlying Evidence Based Investment Selection (EBIS) BN model that computes the expected return and uncertainty of project outcomes under different risk scenarios. We illustrate the power of this approach in planning a large-scale agricultural investment under climate change for an African country. The BN model built for this case study is available at [19].

The remainder of this paper is organized as follows. Section 2 reviews the relevant project selection tools and approaches, Sections 3 presents the proposed methodology and the underlying BN model. Section 4 applies it to evaluate a suite of agricultural investments under different climatic and socio-politic risk factors. Finally, Sections 5 and 6 respectively discuss our results and presents our conclusions.

## 2 Probabilistic models for investment evaluation

Agricultural development investments are inherently complex and uncertain with a poor track record. Data are often scarce, and expert knowledge is used to compensate where data are lacking [7,20]. However, experts are prone to well-known biases when making decisions under uncertainty [21], and decision support models can enable better decisions in this context by helping experts deal with uncertainty and complexity [7,22]. Underlying assumptions of such models should be clear and transparent, as models with an interpretable reasoning mechanism and evidence-base are more likely to be used by decision-makers.

Spreadsheet-based Monte Carlo (MC) simulation models have been a popular approach for computing probabilistic risk assessment primarily due to their ease of implementation. MC approaches have been available for over 60 years and implemented widely for project risk analysis. More recently, MC has also been used for evaluation of agricultural development investments. Wafula et al. [23] used MC to evaluate investment options in honey value chains in Kenya. Lanzanova et al. [24] used this approach to prioritize reservoir protection investments in Burkina Faso. MC simulations repeatedly generate samples for the random variables in the model and makes a statistical analysis of those samples. The main limitation of this approach is backward inference. When a variable is instantiated, the probability distributions of variables on which this variable is conditioned cannot be revised. The structure and conditional probabilities of the model must be transformed in order to make such inference [10,11]. Hence, MC simulation models are often computed by instantiating intermediate variables. Difficulty in understanding modelling assumptions of large MC simulation models is another barrier to their use. Although their modelling assumptions are encoded in spreadsheets, understanding the relations between the model parameters may not be feasible as it may require investigating multiple complex spreadsheets.

Bayesian Networks overcome those limitations of MC simulation models. Firstly, they offer algorithms, such as the Junction Tree (JT) [25], that can compute backward inference, and even abductive inference, without needing to change the representation of model structure and parameters. Secondly, the graphical structure of BN models makes modelling assumptions explicit and more intuitive to interpret.

The graphical structure of a BN is a directed acyclic graph (DAG) composed of nodes representing variables and directed arcs representing relations between those variables [26]. DAG encodes conditional independence assumptions between the variables hence modelling assumptions about the problem domain can be interpreted from the graphical structure. When two nodes are *A* and *B* are directly connected in the BN, as in *A*→*B*, the node with the outgoing arc *A* and incoming arc *B* are respectively called the parent and child of the other. Each node in the BN has an associated conditional probability distribution that defines the strength of the relation with its parents. Hence, a BN represents the joint probability distribution of its nodes based on conditional probability distributions and independence assertions that are encoded in its structure and parameters respectively.

BNs that contain both discrete and continuous variables are called hybrid BNs. Smid et al. [10] and Nash and Hannah [11] argue that the main disadvantage of BNs, compared to MC simulation models, is solving hybrid models. Early BN solving algorithms such as the JT could only solve discrete models, and continuous variables had to be manually discretized. This led to inaccuracies because of the challenge of determining which intervals of the posterior distribution need to have a finer discretization. However, the Dynamic Discretization (DD) algorithm overcomes this limitation by optimizing the discretization for the posterior [27]. The DD algorithm discretizes continuous variables by minimizing an approximate relative entropy error between the true and discretized marginal probability densities. In order to decrease the error, DD divides the discretized states in the high-density areas and combines the states in the zero density areas. After the error is minimized, the discretized model is solved by a discrete inference algorithm such as the JT. The discretization is revised every time a variable is instantiated. DD can compute hybrid BNs with virtually all statistical distributions and deterministic functions of those distributions. Assessment of convergence in DD is simpler compared to alternative sampling-based algorithms for solving hybrid BNs, such as Markov chain Monte Carlo (MCMC). DD has been implemented in AgenaRisk [28] and successfully used in a wide variety of domains [12,13,16,29,30]. Readers are referred to [27] for technical details of the DD algorithm.

In the agriculture domain, BNs have been applied to a wide variety of problems including crop management [31], yield prediction [32], greenhouse gas (GHG) emission [33], farm profitability [34] as well as evaluating policies [35,36]. Readers are referred to [37] for a review of BN applications in agriculture. Several studies have used BNs to evaluate country-wide or regional agricultural policies. Raggi et al. [35] used BNs to assess the impact of agricultural policies in rural areas of Northern Italy. Their model aims to predict whether a farmer intends to exit the sector. Florin et al. [36] used an expert driven BN for a similar task. Their model evaluated the policies for improving farmer engagement with biodiesel production. Richards et al. [38] used BNs to inform policy-makers for developing strategies about how to adapt to climate change. Whitney et al. [39] uses BNs to evaluate the impact of Uganda’s agricultural development policies on nutrition. There studies evaluate higher level policies with qualitative outcome measures, whereas our focus in this study is to evaluate specific investments with monetized financial outcome measures.

Although many BN applications are available, the use of BNs in evaluating agricultural investments have not been widely studied [20]. We have previously proposed a BN framework for making cost, benefit and risk analysis of investment projects [40]. This framework provided a template dynamic BN structure that takes multiple factors into account including investment impact, budget, adoption, and risk events. The use of the framework was illustrated by using generic synthetic examples. However, the framework has several limitations for use as a general project evaluation tool including the following:

1. In order to run the BN model for a specific investment, prior distributions of input distributions in the BN framework have to be defined. This task is not trivial, and no guidelines have been offered. Since the data are scarce in this domain, a variety of resources including online databases, publications and expert knowledge need to be used systematically for defining these parameters.
2. Evaluating and understanding risk factors is a major part of investment evaluation. Risks are, however, modelled in a simple and aggregate way in Yet et al.’s framework [40]. Risks may affect a variety of factors including investment impacts and use of agricultural technologies.
3. Although Yet et al.’s framework [40] has been developed and refined with experts, it has not been applied to a real case. Applications are needed to reveal further requirements and limitations for the framework.

This paper extends and implements the previous work outlined in Yet et al. [40], and aims to address the gaps described above. Firstly, we propose a systematic methodology to define the prior probability distributions required for the BN framework, and discuss the use of different resources for these parameters. Secondly, we improve the risk modelling part of the BN framework. We categorize risks factors according to their impact pathways, and explicitly model these impacts in the BN structure. Finally, we apply the proposed method to a case study. This enables us to review the model structure based on requirements of domain experts, and to assess whether the BN model and prior probability distributions could be applied to a real case with reasonable effort.

## 3 Evidence based investment selection

### 3.1 Overview

**Fig 1:**
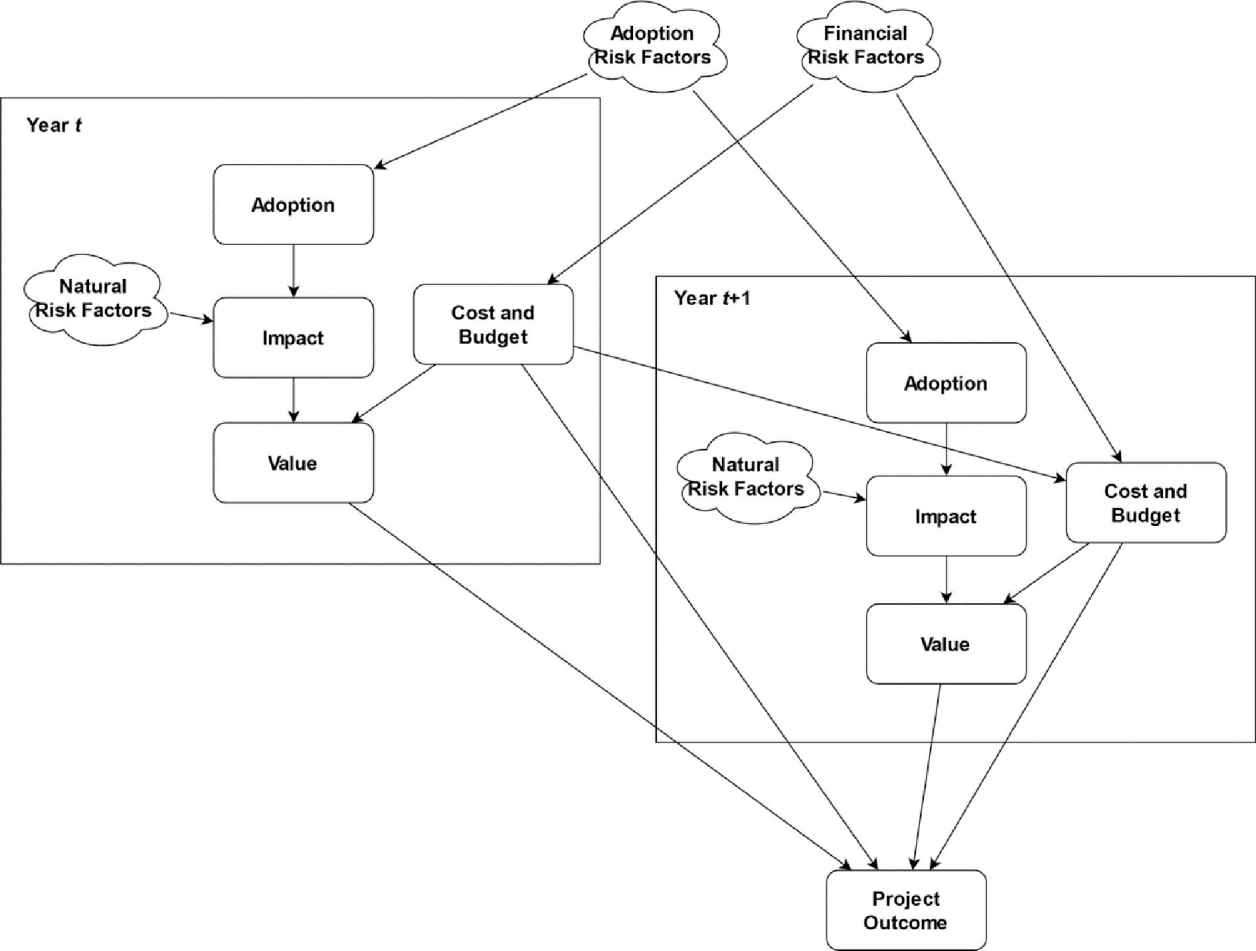
BN overview.

Our method prioritizes investments by predicting their outcomes under multiple risk scenarios by using a BN model. It provides a combined uncertainty and ‘what-if’ sensitivity analysis regarding investment outcomes [41]. We quantify investment outcomes based on widely used financial metrics, including Net Present Value (NPV) and Return on Investment (ROI), and use a BN model to predict NPV and ROI under the uncertainty presented due to variable climate, financial and socio-political situations. We use a dynamic BN structure that models the causal relations between risk factors and financial metrics, and evaluates the project adoption, costs and value over multiple years. The cumulative NPV and ROI distribution of the project are calculated by taking the adoption, impact and costs incur in each year. The model also considers the effect of risk factors to each of those parameters. Hence, what-if analysis and NPV under risk scenarios can be computed. Fig 1 shows an overview of the structure of the BN for two years. BN fragments that represent years are duplicated based on the time horizon of the evaluated investment.

**Fig 2:**
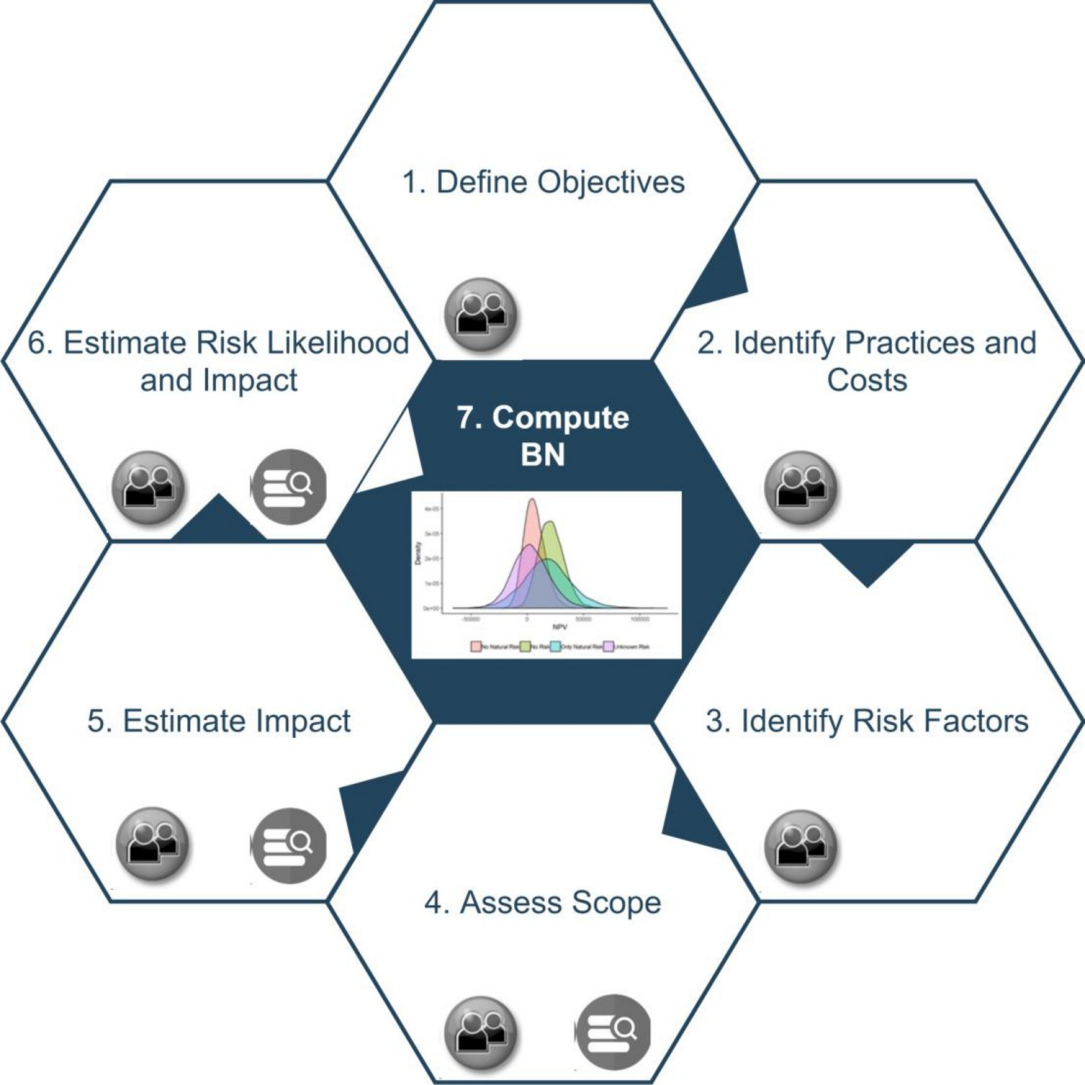
Method overview.

In order to run the BN model, the objectives and scope of the projects being evaluated must be clarified and parameters regarding adoption, impact, cost and risk must be defined. Here, we define a systematic methodology to define these parameters by using a combination of evidence from published evidence and expert knowledge. Fig 2 shows an overview of our method. Our method clarifies the parameters required for populating the BN model with domain experts and identify the relevant evidence for these parameters based on a review of scientific literature and repositories.

### 3.2 Steps of proposed method

Our method is composed of 7 steps:

1. **Define Development Objectives:** Firstly, we clarify the key development objectives of the project. The project evaluation period and overall budget are also defined at this stage.
2. **Identify Relevant Practices and Costs**: Agricultural development projects often implement multiple agricultural practices. For example, a project to improve soil fertility may include practices including using improved fertilizers, fallows and composting practices. We identify all relevant agricultural practices relevant to the project in this step. Yearly project costs are also defined at this stage based on the work required for implementing interventions.
3. **Identify Risk Factors:** Agricultural projects are prone to natural and socio-political risks. A risk factor can affect project impact, adoption or budget in our model. We identify the major risk factors for the project and classify risk factors into three categories according to their effects:
  - Natural risk factors (NRF), such as droughts and floods, may happen in different years and affect productivity and farmer income. Their effect can be different to project beneficiaries and non-adopters.
  - Adoption Risk Factors (ARF) such as political crisis events affect project reach and adoption.
  - Financial Risk Factors (FRF) such as ineffective management affect project budget and costs.
  A risk factor with multiple effect pathways can be classified as multiple categories. Our method and the underlying BN model can be modified to include other risk factor categories.
4. **Assess Scope:** Our model defines the project scope based on the number of people who will directly benefit from the project over the project duration. For agricultural development projects, this includes farmers and their dependants. Census information and domain experts are the primary source of information for estimating this parameter.
5. **Impact Estimates:** Our model monetizes the productivity and climate mitigation impact by estimating the additional income generated for project beneficiaries and using the social cost of carbon respectively. For productivity impact, we define the baseline income distribution for a potential beneficiary before adopting the project, and the distribution of additional income generated by agricultural practices. Baseline income distribution is defined by considering the average income of targeted farmers based on expert knowledge and census information. The change in income data is defined based on review of scientific literature and online repositories such as The Evidence for Resilient Agriculture (ERA), which is a dataset compiled from about 1,400 peer-reviewed publications [42]. ERA contains data on the productivity, mitigation and resilience outcomes of agricultural practices implemented in over 100 farm practices in Africa. In the review, the domain experts identify the data published for each agricultural practice identified in Step 2. If data from the same country are not available, the domain experts focus on data from countries with similar climatic and socioeconomic conditions When multiple estimates are available, meta-analysis is used to combine them. Meta-analysis is an established approach to pool multiple estimates by taking their differences into account [12,43]. Similarly, for the climate mitigation effect, the review aims to estimate the GHG balance of each practice and GHG price.
6. **Define Risk Likelihood and Impact:** Our method explicitly models the likelihood and impact pathway of the risk factors identified in Step 3 in the underlying BN. Hence, parameters regarding the probability and impact of each risk factor must be defined in the BN. A variety of resources can be used and combined for these parameters. The initial resource for these parameters is often expert knowledge. When domain experts define risk factors in Step 3, they can also provide a qualitative estimation of likelihood and effect in an ordinal scale (e.g. low, moderate, high). This is usually the starting point, and, if no other resources are available, these estimations may be directly used in the BN by using ordinal approximation approaches [44].
  Risk likelihoods of natural hazards can be obtained from scientific publications that provides data about previous occurrence of these events [45,46]. The historical data for political risk factors are often limited, and previous data may not be applicable for the current case due to political changes. However, online repositories such as the World Governance Indicator (WGI) [47] rates the political and governmental factors of different countries based on multiple factors. This data could be used as basis for the likelihood estimates of political and financial risk factors.
  We also need to define the impact of risk factors on investment impact, adoption and budget. Moreover, these effects can be different for project beneficiaries and other farmers. For example, the effect of a natural risk factor can be milder for the beneficiaries adopting an improved farming practice compared to non-beneficiaries. Expert knowledge is our primary resource for defining these impacts. Since accurate estimation of these effect can be difficult, we define these effects with intervals that represent the degree of uncertainty of these effects. The BN model enables the use of whole intervals and statistical distributions when computing NPV and ROI. When multiple experts are available, intervals elicited from each expert can be combined [48].
7. **Compute BN:** We finally enter the parameters defined in previous steps to the BN model, and compute the posterior distributions of NPV and ROI over the project duration under different risk scenarios.

### 3.3 BN model

**Fig 3:**
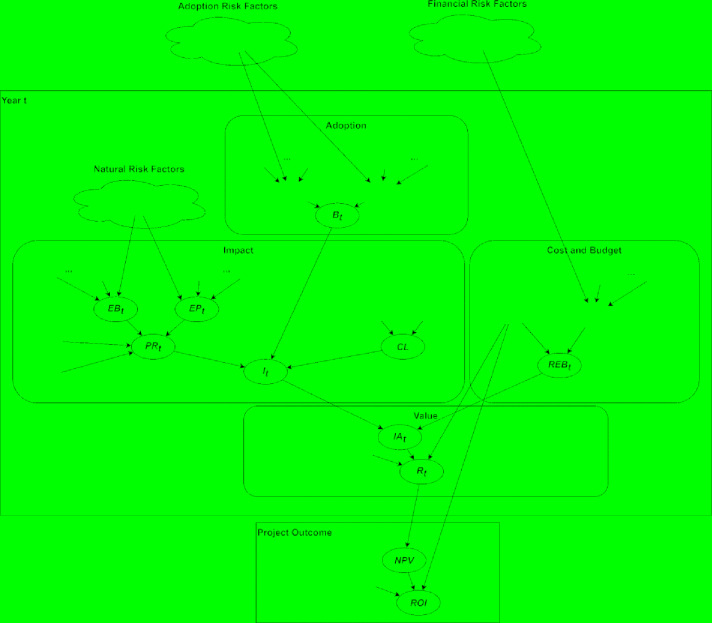
BN fragment for year t.

This section describes each component and variable of the proposed BN model. Fig 3 shows the BN structure corresponding to each BN fragment shown in Fig 1 for year *t*. Oval and square shaped nodes in this figure represent random and deterministic variables respectively. The parameters of grey coloured nodes in Fig 3 need to be defined, based on expert knowledge or data, in order to calculate the BN. Other nodes are determined by functions of their parents. The structure of the BN model is based on widely used financial project evaluation metrics and causal relations of risk factors. Table 1 summarizes the parameters and prior probability distributions required for each component the BN model and shows in which step of the method these parameters are defined. In summary, overall budget, interest rate and project duration are defined in Step 1. The budget is detailed to yearly costs in Step 2. The set of risk factors relevant to the project are identified in Step 3. Total number of beneficiaries and project adoption rate are defined in Step 4. Baseline and beneficiary income for productivity impact, and GHG balance and cost for climate mitigation are defined in Step 5. The probability of each risk factor and their effect on adoption, impact and budget are defined in Step 6. The remainder of this section describes the BN model in more detail.

**Table 1:** Parameters identified in different steps of the proposed method.

Name	Description	Type	Component	Method
*BD*	Project Budget	Continuous	Cost & Budget	Step 1
*d*	Discount Rate	Continuous	Project Value	Step 1
*m*	Evaluation Period	Integer	Project Value	Step 1
*C*_*t*_	Cost Estimate in Year *t*	Continuous	Cost & Budget	Step 2
*TB*	Total Number of Targeted Beneficiaries	Continuous	Adoption	Step 4
*P*	Rate of Innovation	Continuous	Adoption	Step 4
*Q*	Rate of Imitation	Continuous	Adoption	Step 4
*AR*_*t*_	Adoption Rate in Year *t*	Continuous	Adoption	Step 4
*PR*_*j*_	Rate of Innovation under Adoption Risk Factor *j*	Continuous	Adoption	Step 6
*QR*_*j*_	Rate of Imitation under Adoption Risk Factor *j*	Continuous	Adoption	Step 6
*ARR*_*j*, *t*_	Adoption Rate under Adoption Risk Factor *j* in Year *t*	Continuous	Adoption	Step 6
*TBR*_*j*_	Total Number of Targeted Beneficiaries under Adoption Risk Factor *j*	Continuous	Adoption	Step 6
*IB*	Beneficiary Income Before Adopting Project	Continuous	Impact	Step 5
*IP*	Beneficiary Income After Adoption Project	Continuous	Impact	Step 5
*GHGC*	Greenhouse Gas Cost	Continuous	Impact	Step 5
*GHGB*	Greenhouse Gas Balance	Continuous	Impact	Step 5
*EBR*_*i*_	Beneficiary Income Before Project under Natural Risk Factor *i*	Continuous	Impact	Step 6
*EPR*_*i*_	Beneficiary Income After Project under Natural Risk Factor *i*	Continuous	Impact	Step 6
*BDR*_*k*_	Budget under Financial Risk Factor *k*	Continuous	Cost & Budget	Step 6
*ARF*	Adoption Risk Factors	Multinomial	Risk Factors	Step 6
*NRF*_*t*_	Natural Risk Factors in Year *t*	Multinomial	Risk Factors	Step 6
*FRF*	Financial Risk Factors	Multinomial	Risk Factors	Step 6

#### 3.3.1 Risk factors

Risk factors can be modelled as multinomial discrete variables. The BN model contains natural risk factors happening in year *t NRF*_*t*_, adoption risk factors *ARF* and financial risk factors *FRF* that respectively affect the productivity, adoption and budget components of the model. The probability of occurrence for each natural risk factor in year *t* P(*NRF*_t_ = *i*), each adoption risk factor P(*ARF = j*) and financial risk factor P(*FRF = k*) need to be defined. The dependencies between the risk factors and different components of the BN are described in the following sections. Note that, if a risk factor affects multiple components, the BN model can be adapted by adding multiple dependencies in the graphical structure.

#### 3.3.2 Adoption

The BN model defines the project’s scope based on target beneficiaries *TB* i.e. the number of people who will directly benefit from the project over the project duration. For agricultural projects, this includes farmers and their dependants. Target beneficiaries gradually adopt the project over a period of time. Our method offers two alternative ways of defining adoption in the BN model.

Firstly, the ratio of targeted beneficiaries and area that adopts the project in year *t AR*_*t*_. is defined with a probability distribution or an interval that reflect the uncertainty regarding this parameter. The Beta distribution is a suitable statistical distribution for this parameter as it is bounded between 0 and 1.

If adoption risk factors, such as community conflict, are present, adoption rate is modelled as a mixture distribution conditioned on the adoption risk factors in the BN.

$$(AR_{t}|ARF=j)∼{ARR}_{j,t}$$

where *ARR*_*j*,*t*_ is the adoption rate under adoption risk factor *j* in year *t*. The number of beneficiaries that adopts the changes every year due to adoption rate.

$$B_{t}=AR_{t}\times TB$$

where *TB* is the total number of targeted beneficiaries and *B*_*t*_ is the total number of beneficiaries that adopts the project in year *t*.

Some adoption risk factors such as poor governance can directly affect *TB*. In this case, *TB* can be modelled as a mixture distribution conditioned on those factors.

$$(TB|ARF=j)∼T{BR}_{j}$$

where *TBR*_*j*_ is the total number of target beneficiaries under adoption risk factor *j*. Alternatively, the Bass model can also be used in our model to define adoption rates in the BN. The Bass model uses rate of innovation *P* and rate of imitation *Q* to estimate the adoption rate (AR) over a specified time period *t* as shown below:

$$AR_{t}=\frac{1-e^{-(P+Q)t}}{1+(\frac{Q}{P})e^{-(P+Q)t}}$$

If adoption risk factors are present, the rate of innovation and imitation are modelled as mixture distributions conditioned on the risk factors.

$$(P|ARF=j)∼{PR}_{j}$$

$$(Q|ARF=j)∼{QR}_{j}$$

where *PR*_*j*_ and *QR*_*j*_ are respectively the rate of innovation and imitation when the adoption risk factor *j* is present. Although, often no data are available to estimate the Bass model parameters *PR*_*j*_ and *QR*_*j*_ from data, the interpretation of these parameters could be described to domain experts, and parameter intervals could be elicited. However, domain experts may find it difficult to interpret and define *PR*_*j*_ and *QR*_*j*_ parameters especially if they are not familiar with the Bass model. In this case, they may be more comfortable in defining a probability distribution directly for *ARR*_*j*,*t*_.

*Defining scope by total farming area*. In some agricultural development projects, it may be more suitable to define the scope with total farming area rather than total number of beneficiaries. In this case, *TB* and *B*_*t*_ variables are respectively replaced by target area *TA* and total area where the project is implemented in year *t A*_*t*_.

### 3.3.3 Impact

The impact component models the productivity and climate impact of a development project. Productivity impact represents the income difference for a project beneficiary. Climate impact of a project can be modelled by using monetized greenhouse gas (GHG) balance of loss and sequestration. Other environmental impacts beyond climate change were not included in the current model but they could be added similarly to the impact component of the BN. The total impact of the project in year *t*, *i*.*e*. *I*_*t*_, is computed as follows:

$$I_{t}=B_{t}\;{(PR}_{t}+CL)$$

where *PR*_*t*_ and *CL* are respectively the productivity impact per beneficiary in year *t* and climate impact per beneficiary.

*Productivity impact*. Our BN defines the productivity impact based on the difference between the income distribution of farmers before and after adopting the project. Several natural risk factors, such as drought and pests, can affect income. Moreover, the effect of these risk factors could be different for project adopters and non-adopters. For example, while a drought decreases the income of both project beneficiaries and other farmers, its impact can be more severe for the farmers who do not adopt improved practices offered by the investment. In order to model this, we adjust income distributions based on risk factors that realise in different years., *EB*_*t*_ and *EP*_*t*_ be the combined effect of natural risk factors at t. The combined effect of risk factors for project beneficiaries *EP*_*t*_ and other farmers *EB*_*t*_ at *t* are modelled as a mixture distribution conditioned on natural risk factors *NRF*:

$$(EP_{t}|{NRF}_{t}=i)∼E{PR}_{i}$$

$$(EB_{t}|{NRF}_{t}=i)∼E{BR}_{i}$$

where *EPR*_*i*_ and *EBR*_*i*_ are the effect of the natural risk factor i for project beneficiaries and other farmers respectively. Productivity impact for a project beneficiary at year *t PR*_*t*_ is the difference between their income before and after adopting the project adjusted to the combined effect of natural risk factors.

$${PR}_{t}=IP\;EP_{t}-IB\;{EB}_{t}$$

where *IB* and *IP* respectively be the baseline income of a beneficiary before and after adopting the project.

Prior probability distributions for *IB*, *IP*, *EPRi* and *EBRi* need to be defined for the impact component of the BN model. Project location and demographics of potential adopters are considered when defining the *IB*. *IP*, *EPR*_*j*_ and *EBR*_*j*_ can be elicited from domain experts or derived by conducting a literature review and meta-analysis of income impact of relevant agricultural practices and risk factors in under geographical conditions.

*Elicitation of income impact*. It may be easier to elicit or find evidence about the relative impact of a project on farmer incomes *RI* rather than directly eliciting *IP*. In this case, *IP* can be estimated by adjusting *IB* by relative impact as shown below:

IP=IB×RI

*Climate impact*. Climate mitigation impact *CL* of an investment can also be modelled in the BN by considering its GHG balance and the related costs. The three primary GHGs relevant to agricultural production are carbon dioxide (CO_2_), nitrous oxide (N_2_O) and methane (CH_4_). These gases have different relative impact on the climate system, but the effects are put on the same scale by converting them into units of carbon dioxide equivalents (CO_2_-eq). The climate impact of the project is measured by its GHG Balance (GHGB) (measured by t CO_2_-eq/beneficiary/yr) times the cost of t CO_2_-eq in carbon markets i.e. GHG cost (GHGC).

CL=GHGB×GHGC

GHGC can be defined based on the GHG market price. It can be evaluated in different scenarios to reflect the uncertainty of future prices. GHGB can elicited from domain experts or compiled from published data about the mitigation impact of similar agricultural practices.

#### 3.3.4 Cost and budget

The yearly cost estimates *C*_*t*_ of the projects are modelled by probability distributions that represents the degree of uncertainty around these estimates. *C*_*t*_ could be elicited from domain experts at initial stages of the project, or it could be aggregated if detailed costing is available. Cost estimates for later periods can modelled with a higher variance reflecting higher uncertainty regarding long-term estimates. The project costs *C*_*t*_ and budget *BD* can be adjusted due to financial risk factors *FRF*.

$$(BD|FRF=k)∼{BDR}_{k}$$

where *BDR*_*k*_ is the budget available under the financial risk factor *k*.

#### 3.3.5 Value

The adoption, impact and cost estimates computed in previous sections are used to calculate the discounted benefit of the project in each year. Yearly discount or interest rate *d* and the total time period *m* for the project evaluation must be defined in order evaluate the total project value. Over the project duration, the risk of exceeding the budget in year *t*, i.e. *REB*_*t*_ is evaluated by comparing the cumulative cost distribution in that year to the budget *BD*.

$$REB_{t}=\{\begin{array}{l} 1\;if\;(\sum_{i=1}^{t}C_{i}–BD)>0\\ 0\;else \end{array}$$

If the project is over budget in year *t*, the project impact *I*_*t*_ is reduced by the extent of exceeding the budget.

$${IA}_{t}={REB}_{t}\;I_{t}\;RR+(1–{REB}_{t})I_{t}$$

where *IA*_*t*_ is the impact adjusted by the risk of exceeding the budget, *RR* is the reduction rate. The discounted benefit of the project in year *t* is the difference between the adjusted impact and cost discounted by the rate *d*.

$$R_{t}=\frac{\left({IA}_{t}–C_{t}\right)}{{\left(1+d\right)}^{t}}$$

#### 3.3.6 Project outcome

NPV is the sum of discounted benefit over the project duration *m* and ROI is the ratio of NPV over total discounted cost of the project.

$$NPV=\sum_{t=1}^{m}R_{t}$$

$$ROI=\frac{NPV}{\sum_{i=1}^{m}C_{i}}$$

## 4 Case study

In this section, we apply our method to predict the NPV and ROI of an agricultural development project recommended for an African country. The proposed method and the underlying BN were used to prioritize development projects and evaluate their vulnerability risks. Below, we describe how each of these steps and the BN model were applied for this case study.

**Fig 4:**
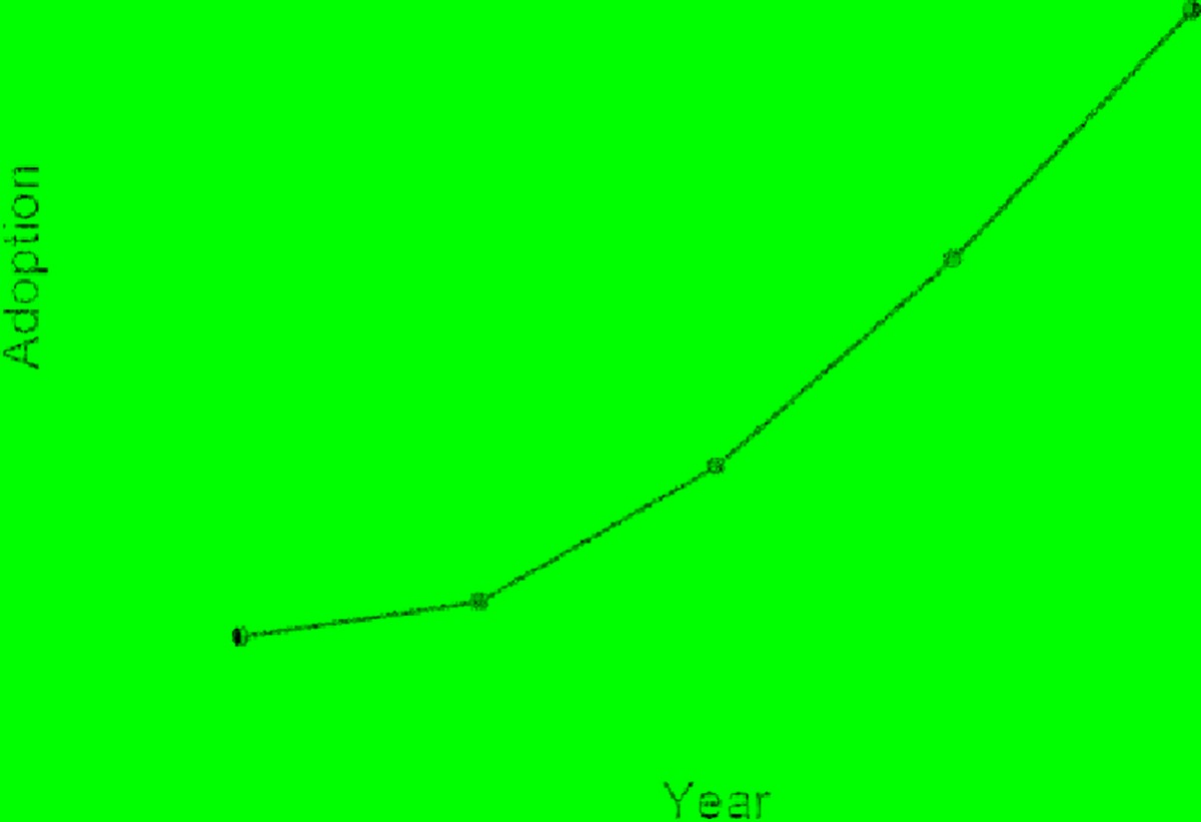
Adoption percentages over project duration.

**Fig 5:**
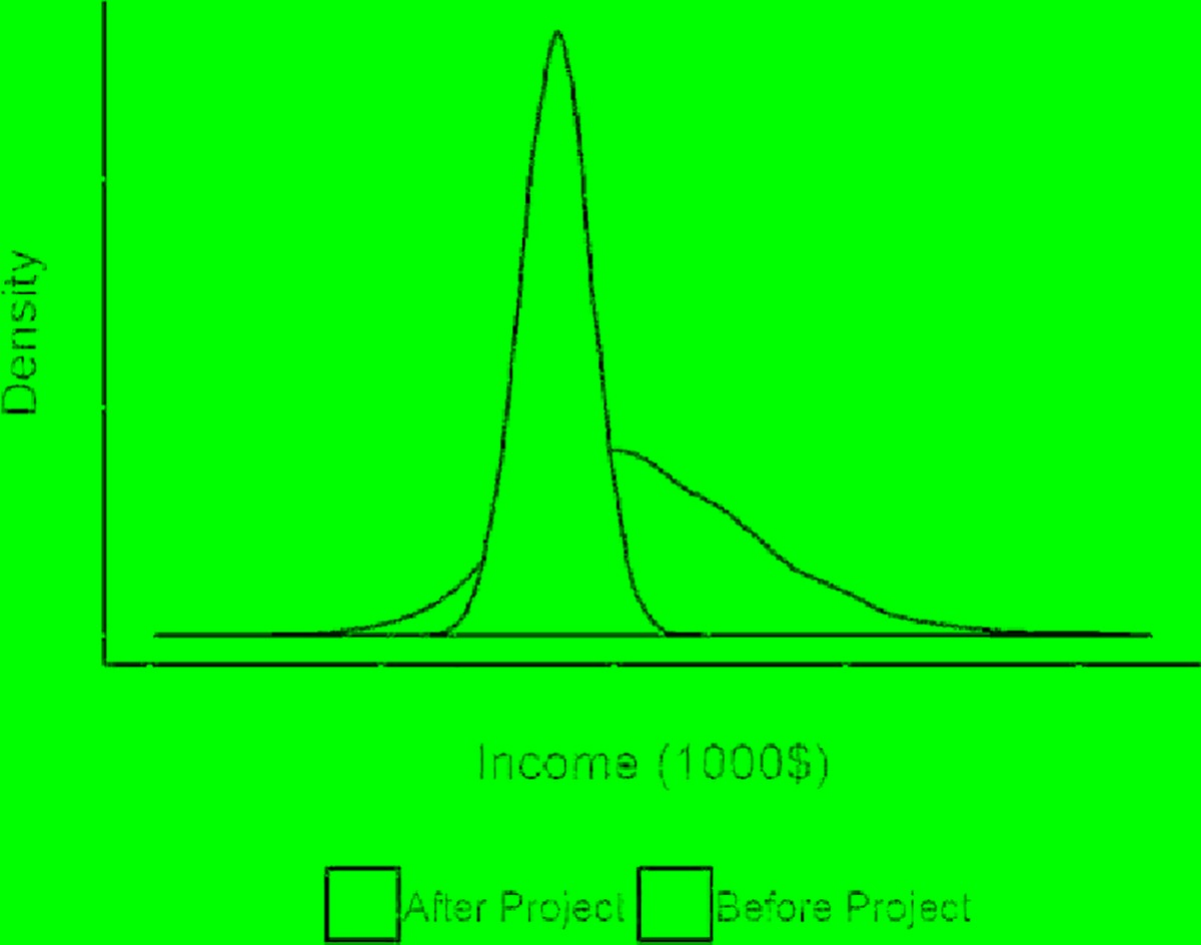
Yearly income distribution before and after adopting project.

1. **Define Development Objectives:** The objectives, duration and budget of the project were detailed in a workshop consisting of a panel of 40 stakeholders including academics, government and civil society representatives. The development objective of this investment is to increase the productivity of agriculture that occurs in the floodplain in the target country to achieve increased food security for smallholder households. The project focuses on cereals, forage for livestock, root crops and vegetables, and it is evaluated for a period of 5 years. The total project budget is 89,000,000 USD.
2. **Identify Interventions:** The farming practices included in this project were also defined in the workshop. The interventions for this project include, use of improved crop varieties, organic and inorganic fertilizers, mulch, and reduced tillage. A costing expert prepared detailed costings of the project for these practices based on input from stakeholders. Table 2 shows the aggregated costs for each year.
3. **Identify Risk Factors**: A preliminary list of risk factors relevant to the project were identified in the workshop. Two domain experts reviewed these risk factors, identified the most critical ones, and categorized them according to their impact pathways. The main risk factors identified for the project are shown below:
  - ***Natural Risk Factors*:** Floods, droughts and pests are the main natural risk factors identified for this project.
  - ***Adoption Risk Factors*:** Political crisis, community conflict and poor governance are the main political risk factors that may affect the adoption and total number of target beneficiaries for this project.
  - ***Financial Risk Factors*:** Donor’s unwillingness to fund the project was identified as a financial risk factor. The domain experts later decided that this risk factor is irrelevant considering the donor’s commitment and reputation to fund development projects.
4. **Assess Scope:** The project interventions are expected to be adopted by 224,000 people in 5 years. The pessimistic and optimistic estimates for this parameter are 168,000 and 280,000 people respectively. The project adoption is expected to be slow, 10% and 30% of the targeted beneficiaries is expected to adopt in Year 2 and 3 (see Fig 4). These numbers are defined based on expert opinion considering the relevant farmer population in the project location, project budget, and complexity and nature of interventions being proposed.
  The prior probabilities for these variables in the BN model were defined based on these estimates. We used the Normal and Beta distributions for the total targeted beneficiaries and adoption rates respectively. The parameters of these distributions can be fitted assuming that optimistic and pessimistic estimates represent their 1^st^ and 99^th^ percentiles.
5. **Estimate Impact:** Baseline income of a beneficiary, before adopting the project, was defined based on expert opinion considering the average income of the farmers where the development project will be done. Income after adopting the project was defined based on the increased output caused by the project. Domain experts analysed data in ERA for the outputs of the farming practices in countries with similar climatic and socioeconomic conditions. Previous data about change yield for cereals, root crops, vegetables and forage for livestock were identified for each practice and combined (see Table 3). The standard deviations of each estimate are shown in parentheses in Table 3. Fig 5 shows the income distributions of potential beneficiaries before and after adopting the projects. Note that, the expected income of beneficiaries is higher than of non-adopters but uncertainty regarding these estimates is high due to the high variation in outcomes of different practices (see Table 3). The uncertainty of these estimates was used in computation of the BN.
6. **Define risk likelihood and effect:** We used a multi-step process to identify risks and estimate likelihood and impact parameters from multiple sources. During the expert workshops, participants generated a list of risk factors associated with the project. After the workshop, two domain experts reviewed the relevant publications and external data repositories to collect statistics and data for the likelihood and impact of these risk factors. External data sources and estimation methodology for each risk factor are detailed below.
  - **Drought:** We estimated drought likelihood from the historic drought frequency in the country over the period 1991–2010 [45]. Drought events were defined as periods where the actual rainfall over the preceding 12 months was more than one standard deviation below the long-term average (Standardized Precipitation Index SPI-12 < -1) based on globally gridded precipitation data. A drought period would begin in a month where the SPI-12 reached -1 and ended in a month where the SPI-12 reached 0, or average rainfall conditions again. The number of such events between 1991–2010 was the reported drought frequency. We calculated mean and variance in number of drought events in each country by dividing the country into 16 grid cells and measuring the mean number of droughts per cell. Drought likelihood was then the average number of drought events per year in the country. For example, if the area averaged 1 drought per decade, then we estimated a 10% chance of drought in any given year.
  - **Floods:** Similar to drought likelihood, we estimated flood likelihood from historic flood data in the country. Reported flood frequency was obtained from the United Nation’s Office for Disaster Risk Reduction’s knowledge platform [49] for the period 2005–2014. Although some flooding occurs annually in the countries during the rainy season, flood events recorded in Prevention Web are Internationally Reported Losses, and thus would be potentially disruptive to project activities. As in drought, flood likelihood was estimated based on the number of observed flood disaster events per year in each country.
  - **Pests:** Data on the frequency of major (and thus project-disrupting) pest outbreaks in Africa are difficult to find. We assessed the likelihood of pest outbreaks using several data sources. In this country, the most common crop pest is the Desert Locust. Locust plagues occurred on 5 occasions between 1900–2000 [50], which yields a conservative estimate of likelihood of 5% in any given year. Additionally, novel or “shock” pest and disease outbreaks have occurred in sub-Saharan Africa approximately 5 times in the past 20 years (Smith 2015), including the most recent outbreak of Fall Army Worm across the continent. This would give a high-end estimate of 25% likelihood of a pest outbreak in any given year. These estimates were combined to yield the final likelihood of a disruptive pest outbreak in any given year.
  - **Political Instability** The country has experienced political crises in the last decade, suggesting a relatively high risk of political instability. To estimate the likelihood of political instability, we used the WGI–Political Stability and Absence of Violence (PSAV) [47]. We converted WGI PSAV scores to likelihood of political instability by establishing a linear scale of 100% chance of instability for a score of -3 (generally those countries in active conflict without functioning governments) and a 0% chance of instability to a score of 2 (the highest given in the dataset). We computed the mean and standard deviation in PSAV score for the country over the 1996–2017 period and converted this to a mean and standard deviation in likelihood of political instability using our linear scale.
  - **Poor Governance:** Similar to Political Instability, we estimated the likelihood of poor governance affecting project implementation using the WGI–Government Effectiveness (GE) [47]. GE Scores were converted to likelihood of poor governance using a linear scale.
  - **Community Conflict:** Community conflict, particularly between agriculturalists and pastoralists or between different ethnic groups, is a potential project risk identified by stakeholders in both countries. We estimated likelihood of community conflict using the Institutional Profiles Database (IPD) 2016 indicators of Social Conflict [51]. The Social Conflict variable includes estimations of ethnic and religious conflict, conflict over land in rural areas, and other types of social conflict. We converted Social Conflict scores to likelihood of conflict using a linear scale, where a score of 0 (serious social conflict) was a 100% chance of conflict and a score of 4 (no social conflict) was a 0% chance of conflict. We used the standard deviation of a country’s scores across the five variables that contribute to the Social Conflict indicator was used to estimate the uncertainty around the likelihood of conflict.
  - **Risk Impacts:** Presence of each political risk factor can cause a 15% - 25% decrease in adoption rates and target beneficiaries. Practices included in the development project offers increased resilience for natural risk factors. Table 1 summarizes the effect of natural risk factors on yearly incomes of project beneficiaries and non-adopters. Note that, in the BN model, political factors affect adoption for the whole project duration, whereas different natural risk factors can happen in different years, and they affect the income in the year they occur. Since the proposed development projects include climate smart practices that provide resilience to climate events, the effect of natural risk factors is milder for project beneficiaries. We used uniform distributions with the bounds shown in Table 4 for the natural risk factors effects in the BN.
7. **Compute BN:** We defined the prior probability distributions in the BN based on the parameters obtained in previous steps. We computed the BN model for each project under four risk scenarios:
  - **No risks:** the probabilities of all risk factors are set to zero.
  - **Natural risks only:** the probabilities of adoption and financial risks are set to zero, non-natural risks may happen.
  - **Political risks only:** the probabilities of natural risks are set to zero, adoption risks may happen.
  - **All risks:** all risk factors may happen.

**Fig 6:**
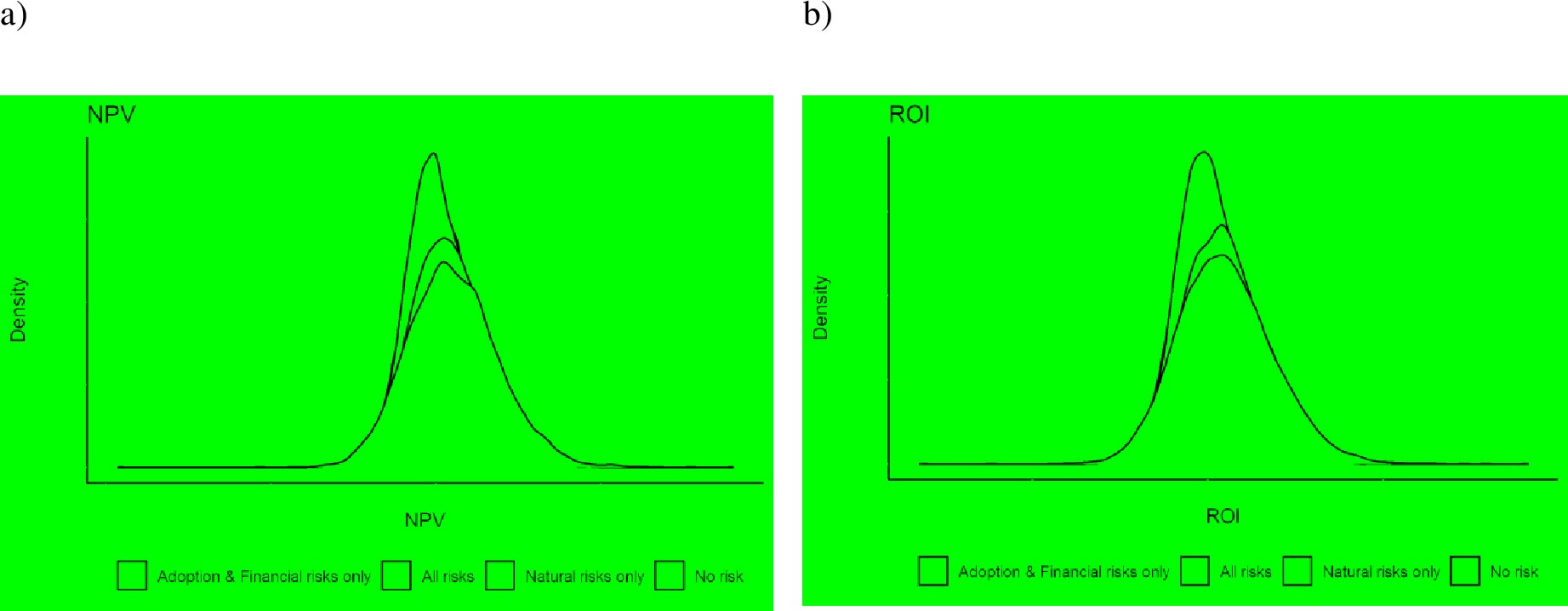
Predicted a) NPV and b) ROI distributions.

Fig 6 shows the NPV and ROI distributions of the project under these scenarios, and Table 5 shows the predicted probability of positive NPV, and the expected values and standard deviations of NPV and ROI. The following section discusses these results and the use of this method.

**Table 2:** Yearly costs for flood recession project.

Year 1	Year 2	Year 3	Year 4	Year 5
38,270,000	11,480,000	13,000,000	9,980,000	8,860,000

**Table 3:** Change in yields.

Practice	%Change Maize	%Change Sorghum	%Change Cassava	%Change Tomato
Improved Varieties	41.3% (40.3%)	69.3% (11.9%)	14.1% (71.7%)	
Inorganic Fertilizer	79.9% (84.8%)	68.0% (73.1%)	23.5% (50.6%)	230.0% (64.6%)
Mulch	51.1% (68.6%)	35.2% (59.6%)	31.7% (34.6%)	47.4% (18.9%)
Organic Fertilizer	59.4% (49.8%)	136.0% (96.0%)	52.1% (66.9%)	157.3% (86.1%)
Reduced Tillage	-8.6% (89.1%)	5.7% (44.1%)	-30.9% (21.6%)	
All Practices	49.6% (60.7)	51.4% (42.4%)	21.0% (42.4%)	76.9% (54.6%)
All Practices–ALL CROPS	46.3% (58.9%)			

**Table 4:** Yearly income adjustment caused by natural risk factors.

Natural Risk Factors	Beneficiaries	Other
Pests	0% - 100%	0% - 80%
Flood	20% - 100%	10% - 70%
Drought	10% - 100%	10% - 70%

**Table 5:** NPV, ROI and probability of positive NPV.

Risk Scenario	NPV (m$ ± SD)	ROI (% ± SD)	% Positive NPV
Flood Recession Agriculture			
No Risks	30.9 ± 82.0	38 ± 100	64
Natural Risks Only	27.5 ± 74.4	33 ± 92	63
Adoption and Financial Risks Only	6.5 ± 62.1	8 ± 77	52
All Risks	4.0 ± 56.5	5 ± 70	50

## 5 Discussion

Without considering risk factors, the project is expected to return 30.9 million USD and 38% of ROI. The probability of a positive NPV is 0.64. The uncertainty around these estimates is high. The standard deviation of NPV and ROI are respectively 82 million USD and 100%, respectively. This is primarily due to uncertainty of yield and income increases caused by the agricultural practices of the investment. The project is resilient to climate risks but vulnerable to social risks. Climate risks cause only a mild decrease in project NPV and ROI. However, political risks cause about 30% decrease in the expected ROI. When all risks are considered, the project only has 0.50 probability of positive return and 5% ROI. Hence, the model currently evaluates the project as a risky investment. It may offer an attractive investment option if political risks could be decreased or mitigated.

The investment alternative evaluated in the case study was an example of a complex decision including multiple agricultural practices and risk factors. Availability of data was too limited to use data-driven approaches for this task. Although various forms of evidence were available for different factors of this decision, evidence had to be reviewed and analysed coherently and consistently for decision-making. Our method offered comprehensive decision support for evaluating the return and uncertainty of the investment under different risk factors. The underlying BN explicitly showed how these parameters relate to each other and how project return is evaluated accordingly in its graphical structure. This enables domain experts to review, and if necessary, change the assumptions underlying the model. Moreover, the parameter uncertainty of all variables and the impact of risk factors were taken into account by the BN when computing the project value. This enables users to assess the uncertainty regarding project NPV and ROI, and make what-if scenario analyses for major risk factors.

A major challenge in using BN models in data-sparse domains such as agricultural development projects is to assign probability distributions to its variables. Although a variety of purely data-driven learning algorithms and evaluation approaches are available for BNs they cannot be used as availability of data is not sufficient to deal with the complexity and uncertainty involved in these domains [7]. Our method provides a structured approach for this task as it uses systematic review of the evidence available in publications and online repositories in consultation with domain experts for those probability distributions. When published evidence or data are not available, probability distributions are based on domain knowledge of experts.

Another challenge in investment evaluation is to compare among projects with different interventions or in different locations. Financial metrics such as NPV enable comparison of dissimilar projects on the same scale. However, it is challenging to use financial models to evaluate non-profit investment projects. The proposed method monetizes commonly accepted evaluation factors for climate smart agricultural projects. These are: productivity, climate mitigation and resilience impact based on additional income generated by investment, GHG balance, and the impact of risk factors. These factors are familiar to most stakeholders and domain experts for agricultural development projects and published evidence is available for them. This enables a suitable framework for defining parameters required for the BN and for comparison of diverse development interventions and projects.

## 6 Conclusion

This paper proposed a methodology for evaluating and prioritizing agricultural investments under different climatic and political risks. Our method is based on a BN model that predicts NPV and ROI. The BN model incorporates the parameter uncertainty of all factors and plays out their implications in its predictions and enables making what-if analysis under risk scenarios. The proposed method combines estimates extracted from multiple publications and elicited from multiple experts for defining the BN parameters as the relevant data are often scarce in this domain. The method was applied to a case-study of evaluating a major agricultural development project in Africa for a financial institution. The method was used to elicit parameters from 15 domain experts and data extracted from scientific publications and ERA.

Agricultural development projects in Africa can be highly risky and uncertain. Therefore, predicting the expected impact of a project is often not sufficient; a richer decision support could be provided by calculating the degree of confidence of the predictions and their resilience to risks. The BN model developed by our method predicts the NPV and ROI of a project and estimates the uncertainty of these predictions under different risk-scenarios. As a result, decision-makers can assess the robustness of projects to different risks and confidence intervals of all model parameters. Moreover, the modelling assumptions are explicitly encoded in a causal structure which could be examined, and modified if necessary, by domain experts. Decision-makers can run a wide variety of scenarios by simply instantiating different variables of this BN.

In the case study, the agricultural development project has a positive expected NPV when all risk factors are considered but this prediction is highly uncertain with just 50% chance of having positive NPV. Even when none of the risk factors are assumed to be present, the NPV predictions still involve uncertainty with 64% chance of positive NPV. This is due to uncertainty involved with other model parameters including adoption rate, scope, costs, and income. The analysis of risk scenarios shows us that the project is more resilient to climate risk factors than to adoption and financial risk factors as the expected value and uncertainty of NPV under climate risk factors is close to the case where no risk factors are considered. Note these results were not evident before we computed the BN model, and a risk and uncertainty analysis to this extent would not be available if we made a typical cost-benefit analysis.

As further research, we intend to develop an online interface for the method that provides a more user-friendly environment for using the proposed method for different development projects. Currently, users need to work with specialized BN software to adapt and compute the BN model for this. The interface will enable users to focus on accuracy of elicitation by automatically handling the BN adjustments and calculations. We also plan to incorporate Value of Information analysis [52] with the proposed method to assist decision makers in prioritizing their data collection efforts.
