# Correction: Evidence-based investment selection: Prioritizing agricultural development investments under climatic and socio-political risk using Bayesian networks

**DOI:** 10.1371/journal.pone.0236909

**Published:** 2020-07-23

**Authors:** The PLOS ONE Staff

Figs 3, 4, 5 and 6 appear incorrectly. The authors have provided corrected versions here. The publisher apologizes for the error.

**Fig 3:**
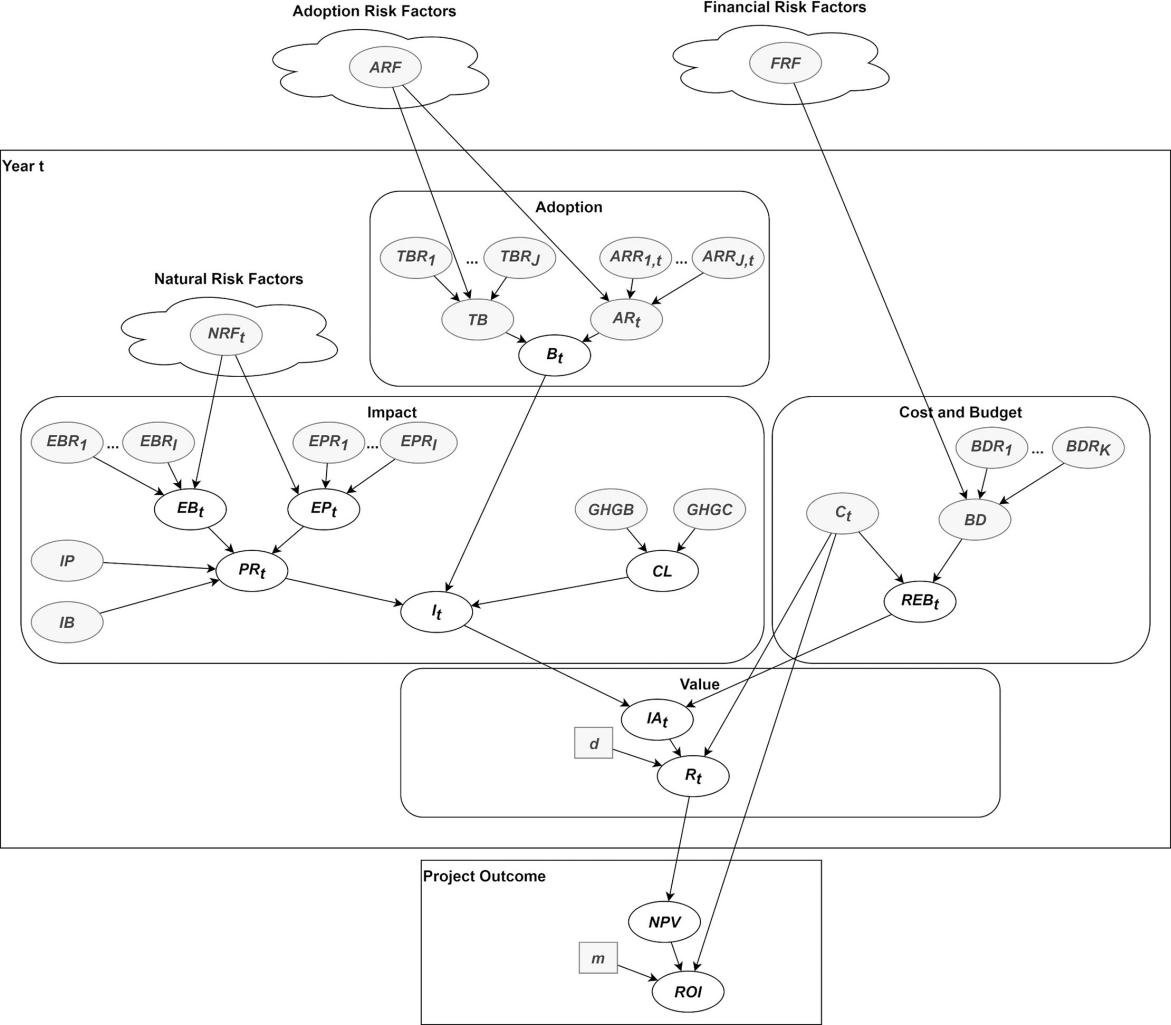
BN fragment for year t.

**Fig 4:**
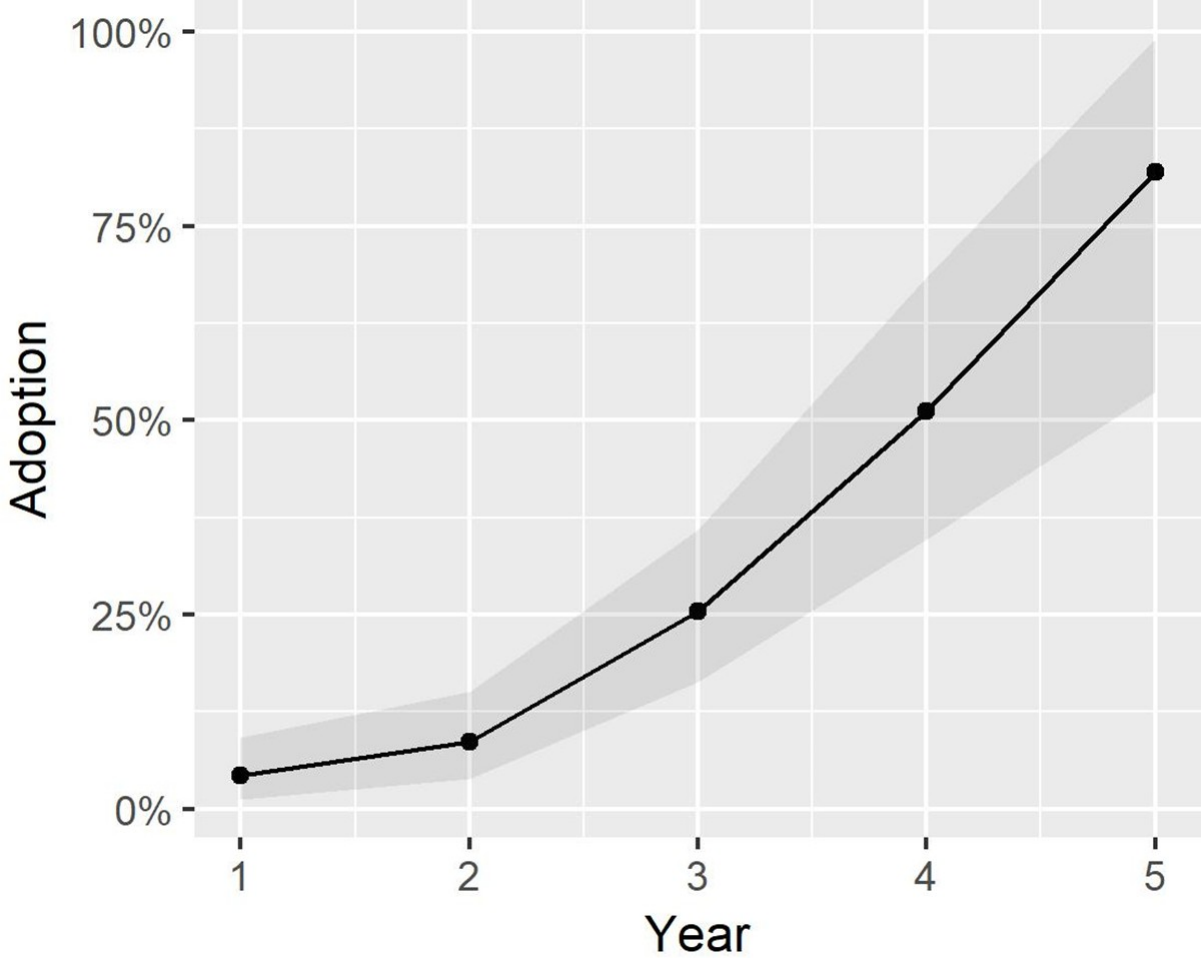
Adoption percentages over project duration.

**Fig 5:**
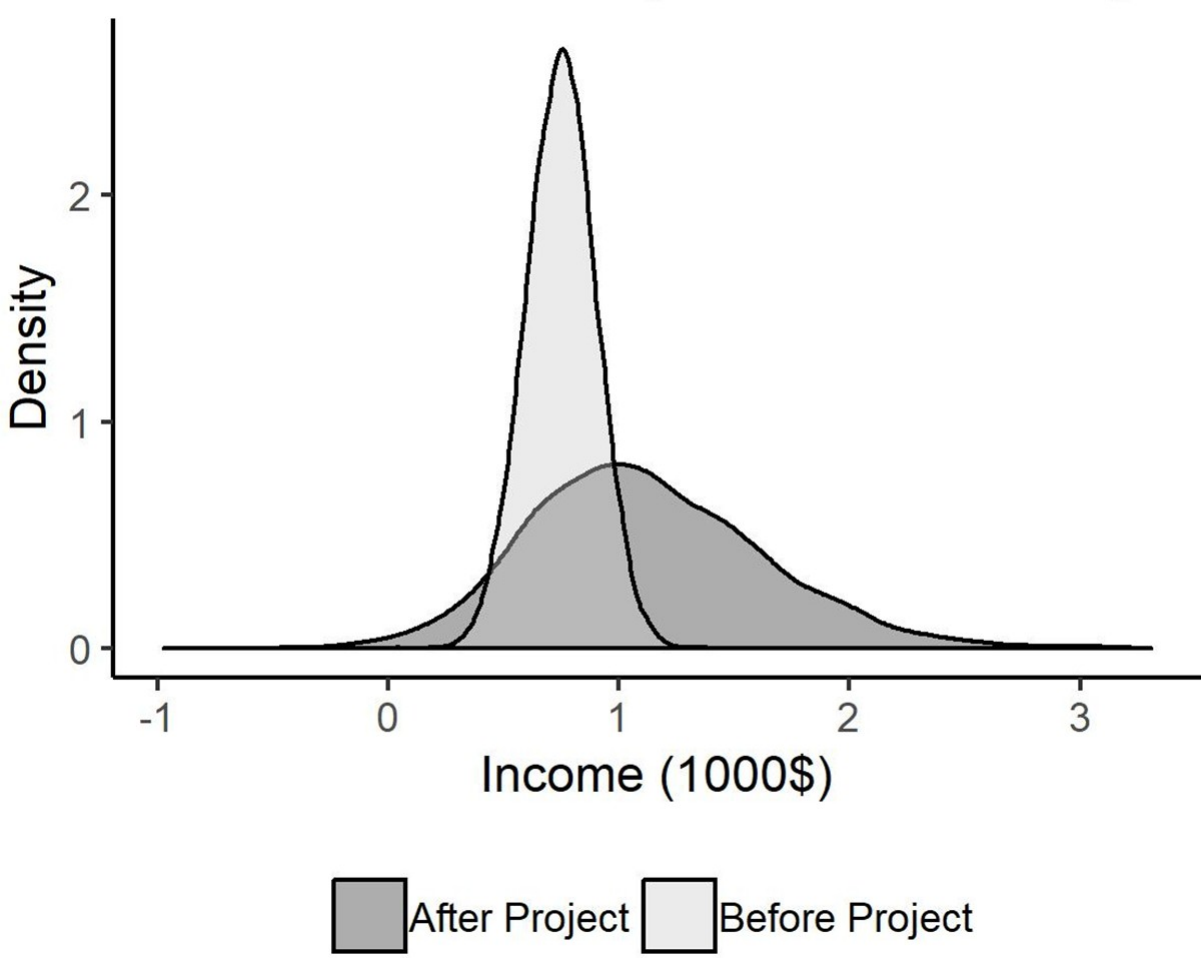
Yearly income distribution before and after adopting project.

**Fig 6:**
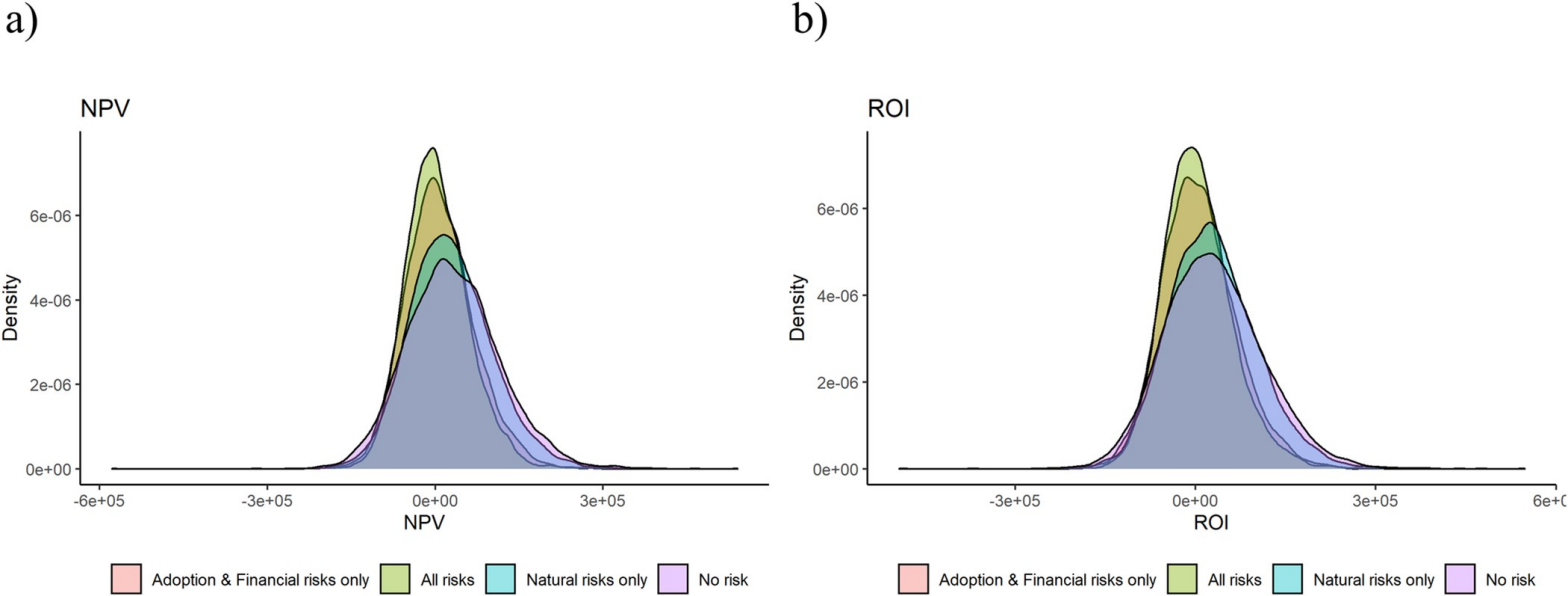
Predicted a) NPV and b) ROI distributions.

The corresponding author’s email address is incorrect. The correct email address for Dr. Barbaros Yet is: barbaros.yet@hacettepe.edu.tr.
